# A feasibility study of a WhatsApp-delivered Transtheoretical Model-based intervention to promote healthy eating habits for firefighters in Hong Kong: a cluster randomized controlled trial

**DOI:** 10.1186/s13063-020-04258-6

**Published:** 2020-06-12

**Authors:** Wing Man Ng, Kin Cheung

**Affiliations:** 1 Division of Science, Engineering and Health Studies, College of Professional and Continuing Education, The Hong Kong Polytechnic University, Hung Hom, Hong Kong; 2 School of Nursing, The Hong Kong Polytechnic University, Hung Hom, Hong Kong

**Keywords:** Healthy eating habits, Health promotion, Body mass index, Fruit and vegetable consumption

## Abstract

**Background:**

Firefighters’ health is often affected by a high prevalence of obesity and cardiovascular diseases, which are common risk factors for sudden cardiac death. The aim of this study is to investigate the feasibility of enhancing healthy eating habits in firefighters through an education programme.

**Methods:**

This will be a cluster randomized control trial study. The participants will be assigned randomly into either control (health promotion pamphlet) or intervention (health promotion pamphlet and education materials through WhatsApp) groups. Changes in healthy eating habits will be assessed by a self-administered questionnaire and anthropometric measurements at three different time points.

**Discussion:**

More education is required in order to improve firefighters’ eating habits.

**Trial registration:**

ISRCTN registry identifier: Registered on 8 April 2019 ISRCTN95472464.

## Background

Obesity and being overweight have many adverse effects on the health of the population in general. Worldwide, in 2012, around 17.6 million people lost their lives due to cardiovascular diseases (CVDs) [1, 2]. Obesity or being overweight is a key factor that can increase the risk of CVDs [3, 4]. A high number of firefighters in the USA, UK and Hong Kong [5–7] have been identified as overweight or obese. Numerous studies have investigated obesity-related CVD risk factors in firefighters, and the results have illustrated this group to be particularly prone to fatal cardiac events [6, 8–12] compared to other professional groups. And yet, the fact is that firefighters need to be in good physical condition, as their work is hazardous. In the USA, approximately 45% of on-duty deaths are caused by sudden cardiac death (SCD) [13–15], with 90% of these events related to coronary heart disease (CHD) [6, 11]. No data have been found to identify any relationship between CVD and obesity in Hong Kong firefighters; however, about 40% have been classified as overweight or obese [16]. Therefore, this issue should be considered seriously.

One study identified that, with nearly 75% of all deaths in the general population globally attributed to CVDs, the major cause was unhealthy eating, such as inadequate intake of fruit and vegetables [17]. This phenomenon is worsening across the world. The long working hours and shift work have an impact on the amount of fruit and vegetables firefighters eat [18]. Their working conditions are unique, as they have 24-h work shifts, are in a quasi-military working organization and the pattern of generally routine firehouse-based activities is interrupted by unpredictable calls for emergency care [19]. Furthermore, firefighters are inclined to eat quickly while on duty, and sometimes their meals are interrupted by fire emergencies. All of these factors can combine to cause them to consume inadequate amounts of fruit and vegetables [20].

Several studies have found that increasing fruit and vegetable intakes can help to minimize the risk of obesity and CVDs [21–23]. In fact, some studies [24, 25] have identified a negative relationship between low consumption of fruit and vegetables and obesity in Asians, Native Hawaiians/Pacific Islanders and Caucasians. In the USA and Hong Kong, large numbers of firefighters have been found to consume less than the recommended five servings of fruit and vegetables per day [26–28]. It is necessary to explore ways to reduce the risk of obesity and CVDs in firefighters by encouraging them to increase their fruit and vegetable intake. One way of doing so may be to develop an effective strategy for promoting healthy eating.

Healthy eating and physical activity are well-known strategies for reducing the risk of CVDs and promoting health benefits [12, 13, 24, 29]. As explained above, even though firefighters concentrate on physical training, they often overlook healthy eating [6, 7]. Exercise has been found to have only a minimal effect as the first interventional option for managing the weight problems of most overweight or obese people [30]. Furthermore, healthy eating is a modifying factor and can also be a key component to minimize the risk of CVDs. Healthy eating should be promoted, as evidence has shown a positive correlation effect between increased consumption of fruit and vegetables and decreased intake of unhealthy foods in various age groups [31–36].

Several studies have demonstrated the positive effects of health education programmes on healthy eating and the reduction of obesity for various populations [37–41]. Traditionally, information about healthy eating has been delivered through seminars, educational videotapes and pamphlets. However, these delivery methods have limitations, including the long durations of seminars or their restriction to specific venues [13, 42, 43]. It may not be suitable for firefighters to attend lengthy seminars in specific locations, as they work according to unique and special roster patterns that are unpredictable. Therefore, the traditional methods of promoting healthy eating are not always appropriate for firefighters.

Furthermore, there may be a gender factor to be considered. Past studies have demonstrated that males and females have different eating habits. This is due at least partly to differences in body size [44]. Gough and Conner (2006) discovered that females were more aware of healthy eating than were males [17, 45]. Food preferences and eating styles have also been found to be gender-related [46]. Since most firefighters are males [47–49], it is vital to understand more about how to improve the eating habits of male firefighters. Since many firefighters work on either “24 hours on, 48 hours off” or “48 consecutive hours followed by 96 hours off” rotations [50–53], they tend to live and work in their fire stations for 24 or 48 h per shift, eating their meals and spending their leisure time together. Some studies have found that, in general, eating habits are affected strongly by social context [54] and are also influenced by peers, especially away from home [55–57]. Furthermore, it has been found that eating habits are different when people have their meals alone or with peers [54]. In order to minimize any potential bias, the peer influence should be controlled.

The interruptions by unforeseeable calls for emergency care [19], along with the other factors described above, mean that firefighters cannot engage in regular programmes to promote healthy eating. This suggests that flexible delivery methods are needed to facilitate them to join a health promotion programme. Social networking can be a good platform for sharing health-related articles and discussing ideas [58]. Many people consider WhatsApp to be a major messaging service [59]. WhatsApp is a mobile instant messaging application that offers real-time texting or communication [60]. The messages can be retrieved even if the users are offline, out of network coverage or when their devices are switched off when a message is sent, and users can join in the WhatsApp at any time [61]. WhatsApp can be a flexible and effective delivery method for a programme such as health promotion for a group requiring flexible delivery, as firefighters do. The results of several healthy eating promotion programmes delivered through WhatsApp have shown that diverse populations increased their fruit and vegetable consumption, reduced their waist circumferences and decreased their intake of unhealthy snacks [62–65]. Based on the above evidence, WhatsApp has been identified as a social networking platform to promote healthy eating as a way to minimize the risk of obesity and its related adverse outcomes in firefighters.

Several studies have identified the need to identify and implement a theory-based intervention as an effective way to promote behavioral change [66–68]. However, there have been very few theoretically based studies on the promotion of healthy eating habits [66]. The Transtheoretical Model (TTM) has been used to modify risky habits such as smoking, lack of physical activity and unhealthy diets [69–75]. The TTM is an effective theory for motivating changes in eating behaviours in diverse adult populations [25, 76–79]. The TTM consists of a series of five distinct stages of readiness for behavioural change, namely precontemplation, contemplation, preparation, action and maintenance stages [80], based on the cognitive, motivational and behavioural aspects of modifying lifestyle habits [81–83]. This model has been adopted widely to promote healthy eating in different populations [43, 71, 84–88]. One study reported positive results for increasing the intake of fruit and vegetables through applying the TTM to the promotion of healthy eating [89].

The application of TTM has been found to have positive effects on healthy eating and changes to eating habits [43, 87, 90–93]. Additionally, numerous studies have found that baseline stage-matched interventions, matched to the participants’ stages at the baseline (for example, for a participant who is at the precontemplation stage, intervention will also be started at the precontemplation stage), can lead to long-term alterations in the dietary habits of overweight adults [94, 95]. One study recommended that stage matching should be considered when designing education about fruit and vegetable intake [96]. Therefore, a baseline stage-matched intervention could be a worthwhile way to promote healthy eating and encourage participants to change their eating habits [78, 97].

### Research gap

Until now, no other tailor-made health promotion programmes have been found to be effective for male firefighters in Hong Kong, who are precluded from existing programmes due to the nature of their work. There is potential to address this gap by the use of WhatsApp as a vehicle for improving healthy eating habits. Since no previous studies have attempted to do this from a theoretical base, this will be the focus of the current study.

### Study aims

This study has two aims. The first is to determine the feasibility of using a TTM-based printed promotion pamphlet and delivering it with stage-matched teaching materials through WhatsApp to promote healthy eating. The second is to evaluate the potential effects of WhatsApp as a vehicle to promote healthy eating habits in firefighters in Hong Kong. The desired primary outcome is changed eating habits, while the secondary outcomes are body mass index (BMI) and waist-to-hip ratio (WHR).

The following research questions have been set: (1) Will the use of the TTM-based promotion pamphlet and WhatsApp bring about greater changes in their eating habits than the use of the TTM-based pamphlet alone? (2) Will the use of the TTM-based promotion pamphlet and WhatsApp lead to greater changes in the firefighters’ BMIs than the use of the TTM-based pamphlet alone? (3) Will the use of the TTM-based promotion pamphlet and WhatsApp change the firefighters’ WHRs more than the use of the TTM-based pamphlet alone?

In order to address two of the “areas of focus” of a feasibility study (Implementation and Practicality), two more research questions have been posed: If the use of WhatsApp helps to improve firefighters’ eating habits, will this also reduce their BMIs and WHRs and increase their fruit and vegetable intake? Will firefighters find this intervention more useful and efficient than the TTM-based promotion pamphlet?

## Methods/design

### Study design

**Fig. 1:**
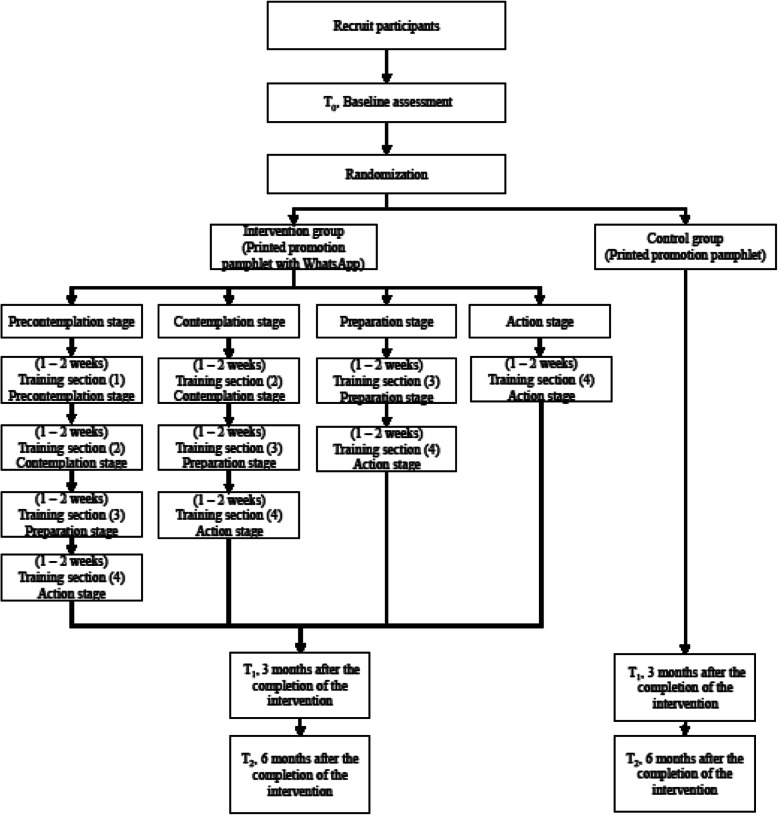
Study design

A two-armed, pre-post test, clustered randomized control trial (CRCT) design will be used in this study. Figure 1 shows the overall design. The data will be collected at three time points: T_0_, baseline; T_1_, 3 months after the completion of an 8-week intervention; and T_2_, 6 months after the intervention.

### Participants

The participants, firefighting teams’ members from the Hong Kong Fire Service Department (HKFSD), will be recruited from fire stations during their free time by the distribution of promotional pamphlets to firefighters in their corresponding fire stations through their fire station colleagues. When a potential participant expresses his interest to participate in this study, he will receive a detailed information sheet (Additional file 1), consent form (Additional file 2) and baseline questionnaire (Additional file 3). Each fire station will be treated as an individual cluster.

### Inclusion criteria

Inclusion criteria will be: (1) male; (2) aged 18 years or older; (3) currently working for the HKFSD as firefighter, working on “24 hours on, 48 hours off” shift; and (4) ownerof smartphone with Internet access. Firefighters who are participating in any other relevant health promotion programmes at the time of the study will be excluded. Written informed consent (Additional file 2) will be obtained from each participant. In order to avoid interference or contamination of data, all participants will be reminded not to disclose any information which they have received from the researcher, and this reminder will be stated in the consent form.

### Recruitment

The participants will be recruited from more than one fire station, located in different districts in Hong Kong. Convenience and snowball sampling methods will be used for the recruitment. The associated participants will be assigned randomly into either intervention or control groups. All the associated participants will be blinded to their intervention allocation.

### Sample size

There are no clear definitions or guidelines for estimating the sample size for a feasibility study [98]. According to [99], a sample size of 10–15 in a group will probably be sufficient. The proposed sample size was calculated to be 38, allowing for an estimation of the feasibility proportions of adherence and retention to within at least ±17% [100, 101] using a 95% confidence interval with a power of 80% to detect an effect size of 0.6 [77]. The main purposes of this exploratory feasibility study are to collect data to allow the design and sample size calculation for the CRCT.

### Teaching materials

The teaching material content has already been designed on the basis of three sources: Promoting Healthy Lifestyles: Alternative Models’ Effects (PHLAME) [102], the Centre for Food Safety [103] and the Department of Health in Hong Kong [104]. In order to design an intervention on healthy eating which would be suitable for Hong Kong firefighters, existing information about healthy eating education and four out of ten sessions which focused on healthy eating from PHLAME have been modified. The teaching materials focus on (1) the rationales for healthy eating; (2) the advantages of consuming fruit and vegetables; (3) understanding methods of cooking fruit and vegetables; and (4) practical tips for getting enough fruit and vegetables when eating out and during festival seasons. The TTM was also designed to assess each intervention participant’s “stage of change”. The TTM includes four core constructs: stages of change, processes of change, decisional balance and self-efficacy [105]. In formulating this intervention, guidelines such as goals, processes of change, strategies and health promotion information (HPI) given through WhatsApp were set with reference to those developed by Lee et al. (2017) to support the application of TTM to promote healthy eating [106]. These teaching materials will be delivered to the participants in the intervention group through WhatsApp.

### Development of pamphlet

The contents of the pamphlet will be the same as the teaching material, as described above. All participants will receive all stages of the intervention, regardless of which stage they belong to at the baseline.

### Fidelity of pamphlet

The fidelity of the teaching materials and pamphlet have been established by a panel of experts including three experienced registered nurses, one dietitian and two nutritionists. All of them have more than 10 years of experience in their own areas of expertise. A checklist was completed by each expert. The checklist indicates all components and aspects of the intervention that the participants will receive, from precontemplation to action stages. Self-report measures of consistency will be in binomial (yes/no) and ordinal (1 = Not relevant, 2 = Somewhat relevant, 3 = Quite relevant, 4 = Highly relevant) formats in multiple item surveys. The advantages of using this method are the low cost, ease of administration and speed of data collection. All experts agreed that the proposed teaching materials should include HPI items with stage-based TTM given through WhatsApp. However, one of the experts did not agree with the others on the content of one item. All the items related to goals, processes, strategies and HPI were rated either “Quite relevant” or “Highly relevant”.

### Development of questionnaire

A questionnaire (Additional file 3) has been developed on the basis of several studies [26, 27, 107–110]. This consists of six study aspects: (1) Personal information; (2) Working characteristics; (3) Eating habits; (4) Health promotion programme for healthy eating; (5) Stage of change; and (6) Decisional balance and self-efficacy.

The questionnaire includes 11 items to assess the Hong Kong firefighters’ eating habits, especially their fruit and vegetable consumption. These questions are based on a series of eating habit studies [26, 27, 107, 108]. The consumption of fruit and vegetables will be measured by asking: (1) “How often did you consume fruits/vegetables in the past week?” (responses ranging from “Not consumed” to “Seven days”); (2) “Where do you usually have fruits/vegetables?” (three choices are “At home”, “During duty” or “No difference, at home and during duty”); and (3) “On a day you consumed the fruits/vegetables, how much did you take on average on that day?” (in bowls or servings). For eating habits, the questions also included “On average, how many days do you have breakfast/lunch/dinner/night snack (take away included) within a week?” (responses ranging from “None” to “More than 5 days”) and “How about the speed of having meals when you are on duty in the fire station when compared to the meals you eat at home?” (responses ranging from “Slower” to “Faster”).

Thirty-five questions were designed to understand decisional balance. Based on Ma et al. (2003) [110], they address the pros, cons and self-efficacy of eating fruit and vegetables. The decisional balance will be evaluated by asking the participants how important each of the pros and cons is in their decisions to consume recommended amounts of fruit or vegetables, using 5-point Likert scales ranging from 0 (Not at all important) to 5 (Very important). Eight pros and nine cons for fruit intake and eight pros and ten cons for vegetable intake will be used to assess the decisional balance.

Twelve questions were designed to assess self-efficacy by rating, on 5-point Likert scales ranging from 0 (Very difficult) to 5 (Very easy), how difficult or easy the participants find it to eat based on the recommendations in six high-risk situations, for each of the two dietary habits [109].

Four questions were designed to identify the stages of change in eating fruit and vegetables (i.e. “How many servings of fruits/vegetables the respondent usually consumed each day; intention to eat ≥ 2 servings a day of fruit or ≥ 3 or more servings a day of vegetables; whether the participant had been consuming ≥ 2 servings of fruit or ≥ 3 servings of vegetables for more than 6 months; intention to eat more). The participants will then be asked to state their intentions to have servings of fruit and vegetables by choosing one of five statements, each representing a stage of change: “No, and I do not intend to in the next 6 months” [Precontemplation]; “No, but I intend to in the next 6 months” [Contemplation]; “No, but I intend to in the next 30 days” [Preparation]; “Yes, I have been doing so for less than 6 months [Action]; Yes, I have been doing so for more than 6 months [Maintenance]. These four questions and their related intentions were drawn from De Vet et al. (2006) [109].

Another three questions were developed to assess the Hong Kong firefighters’ points of view about the healthy eating promotion: (1) “Can a healthy eating promotion programme help you to change or understand your eating attitudes and habits?”; (2) “Can a pamphlet on healthy eating alone facilitate you to change or understand your eating attitudes and habits?”; (3) “Can a healthy eating promotion programme delivered through a mobile app facilitate you to change or understand your eating attitudes and habits?” The firefighters will be required to rate these three questions on 4-point Likert scales ranging from 1 “Absolutely cannot” to 4 “Absolutely can”.

### Validity of questionnaire

A panel of six experts, three nursing professionals, one dietitian and two nutritionists was invited to establish the content validity for the questionnaire. The content validity index (CVI) was 0.966, with average item CVIs ranging from 0.667 to 1.000 and individual panel members’ CVIs ranging from 0.885 to 0.987. These results indicate that the questionnaire is valid to investigate the firefighters’ eating habits. The original English version of the questionnaire was translated into Chinese by a professional translator, and another professional translator performed back-translation, obtaining a CVI of no less than 0.8. The reliability of this questionnaire was examined using test-retest reliability. Ten firefighters from the HKFSD, who fulfilled the inclusion criteria for the study, were invited to take part in this reliability test. Their healthy eating habits was examined on day 1 and day 15 (2 weeks in between) using this questionnaire. The agreement between the data collected on these 2 days ranged from 0.704 to 1.00, with a mean of 0.75. All of these correlation coefficients were significant at the 0.01 level.

### Body measurements

Anthropometric data will be the secondary outcome of this study (Additional file 4). The participants will be weighed wearing light clothing and no shoes. Body weight will be measured by the researcher using an Innocare weighting scale. When measuring body height, a Butterfly Brand measuring tape and a 10-in. triangular ruler will be placed perpendicular to the wall to mark the top of the participant’s head, and a Butterfly Brand measuring tape will then be used to measure the distance along the wall from the floor to the ruler. The BMI (in kilograms/meters squared) will be calculated from height and weight measurements. Waist circumference and hip circumference will be measured by the researcher, using the Butterfly Brand measuring tape.

In order to ensure the reliability of the data collected by the researcher, a professional nurse will be invited to perform the data collection alongside the researcher to ensure inter-rater and intra-rater reliability. The measurement will be performed by the researcher and the professional nurse. The agreements between the professional nurse and the researcher should keep the intraclass correlation coefficient (ICC) at no less than 0.8 [111–113] for each measurement.

### Randomization

The participating firefighters who fulfil the inclusion criteria are stratified into different clusters on the basis of the location of their fire stations prior to the randomization process. These clusters will be randomly assigned to be either intervention or control groups by using a simple randomization method which involves a set of random numbers generated by a computer. Odd and even numbers will represent the control and intervention groups respectively. In order to minimize the bias in this randomization, this process will be conducted by an information technology (IT) expert who is independent of the research team and has more than 15 years’ experience in this field. Furthermore, this IT expert will be blinded during this process. All the participants will be blinded as well, which means they will not know whether they are in the intervention or control groups.

### Data collection

**Fig. 2:**
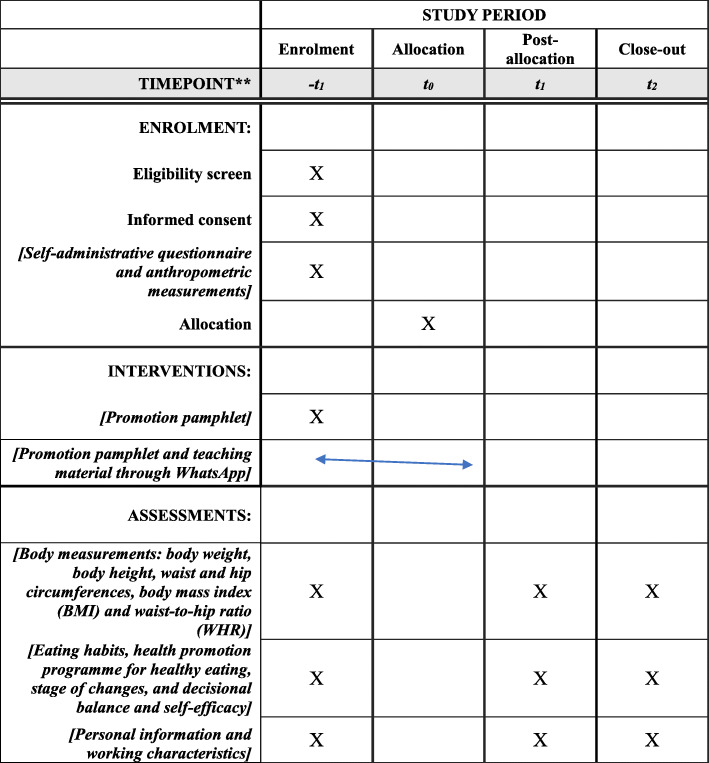
Schedule of enrolment, interventions and assessments

Ethical approval has been obtained from the Human Subjects Ethics Sub-committee (HSESC) [HSEARS20180527001] of the Hong Kong Polytechnic University. An information (study aims, procedure and duration) sheet (Additional file 1) will be provided, and a signed consent form (Additional file 2) will be obtained from each participant prior to the data collection by the researcher. The baseline questionnaire (Additional file 3) will then be completed by the participants, and anthropometric data including body height, body weight, waist circumference, hip circumference, BMI and WHR will be measured (Additional file 4). The data will be collected by the researcher in either a University laboratory or the participants’ fire stations at three different time points: T_0_ (baseline), T_1_ (3 months after the completion of the 8-week intervention) and T_2_ (6 months after the completion of the intervention). The participants will be reminded about the follow-up data collection 1 month and 2 days in advance through WhatsApp. All the data will be accessed by the research team only. The data will be stored on the study team’s password-protected computer and external hard disk. Only study team members will be able to access the data. The researcher will input participant data from hard copy questionnaires into statistical software, Statistical Package for the Social Sciences (SPSS; SPSS Inc., Chicago, IL, USA), within 2 weeks after the data collection. The hard copy questionnaires will be placed in the locker. The schedule of enrolment, interventions and assessments is shown in Fig. 2.

#### Intervention group

The participants in the intervention group will be required to complete the questionnaire, and their anthropometric data will be recorded. Then they will receive the pamphlet from the researcher at T_0_ and the baseline stage-matched teaching material every 2 weeks through WhatsApp.

#### Control group

Similarly, the participants in the control group will also be required to complete the questionnaire, and their anthropometric data will be recorded. The only difference between the intervention and control groups is that the participants in the control group will receive the pamphlet (the same as for the intervention group) from the researcher at T_0_ only.

### Data analysis

All of the collected data will be cleaned prior to the analysis process. The data analysis will be conducted by a statistician who is blind to the participants’ group allocation. The participants’ demographic characteristics will be presented using descriptive statistics (mean and standard deviation) for the continuous data, including age, body height and weight plus BMI, and percentage frequencies for the categorical data, including gender, marital status and educational level. Inter-relationships between the variables will be assessed using Pearson’s correlation coefficient. Differences between the intervention and control groups on outcome indicators will be compared using *t* tests and chi-square tests for the continuous and categorical data respectively. The effect of time (baseline; 3 and 6 months after the completion of the programme) on the outcome measurements will be investigated using repeated measures analysis of variance (ANOVA). The impact of “baseline stage matching” on “stage of change” and other outcome measures within the intervention group will be compared using the Kruskal-Wallis *H* test and one-way ANOVA. Normality tests will be conducted. If the data are normally distributed, it will be appropriate to use parametric tests such as one-way ANOVA. But if they are not normally distributed, non-parametric tests such as the Kruskal-Wallis *H* test will be used. *P* values of less than 0.01 will be considered as statistically significant for all comparisons. All statistical analyses will be conducted using SPSS version 22.0. Intention-to-treat analyses will be applied. As of now, there is no clear cut-off for the acceptable proportion of missing data in a dataset for statistical data analysis. Questionnaires with missing rates of more than 10% will be regarded as “disqualified” and excluded from the analysis. Various statistical methods will be used to treat missing data, including replacement by means or values from the regression analyses, depending on the amount and type of missing data.

## Discussion

It is essential for Hong Kong firefighters to improve and sustain healthy eating habits . The findings of this study will provide the foundation for a bigger research project, which can lead to a longitudinal study to observe the sustainability of firefighters’ healthy eating habits. Furthermore, during the entire research project, we will recruit firefighters continually, to see if we can identify any intrinsic factors to influence their eating habits. This clustered randomized control trial study will make an important contribution to the study of the effectiveness of health promotion programmes for firefighters.

### Trial status

Trial recruitment took place from 1 September 2019 to 30 October 2019 using protocol version 3 dated 17 July 2019. Completed on 29 Feb 2020.

## Supplementary information

**Additional file 1:** Information sheet.

**Additional file 2:** Informed consent form.

**Additional file 3:** Self-administered questionnaire.

**Additional file 4:** Record of measurements.

## Data Availability

The datasets used during the current study will be available from the corresponding author on reasonable request. Materials including educational pamphlets and questionnaires will be available upon reasonable request.

## Abbreviations

ANOVA: Analysis of variance
BMI: Body mass index
CRCT: Clustered randomized controlled trialCVI Content validity index
ICC: Intraclass correlation coefficient
PHLAME: Promoting Healthy Lifestyles: Alternative Models’ Effects
WHR: Waist-to-hip ratio
