# Supplementary material for: A feasibility study of a WhatsApp-delivered Transtheoretical Model-based intervention to promote healthy eating habits for firefighters in Hong Kong: a cluster randomized controlled trial
Source: Trials. 2020 Jun 12;21:518. doi: 10.1186/s13063-020-04258-6 (PMC7291567; doi:10.1186/s13063-020-04258-6)
Supplement: Supplementary file 1 — Additional file 1: Information sheet. [file 13063_2020_4258_MOESM1_ESM.pdf]

香港理工大學
THE HONG KONG
POLYTECHNIC UNIVERSITY

醫療及社會科學院
Faculty of Health and Social Sciences

香港 九龍 紅磡
Hung Hom Kowloon Hong Kong

INFORMATION SHEET

A feasibility study of using various channels to promote healthy eating habits for firefighters in Hong Kong: A cluster randomized controlled trial

You are invited to participate on a study supervised by Dr. Kin Cheung, and conducted by Ms. Ng Wing Man, who is post-graduate student of the Faculty of Health and Social Sciences in The Hong Kong Polytechnic University.

The aim of this study is to promote healthy eating using various channels in order to enhance Hong Kong firefighters' eating habits. So that, your participation is very important indeed.

According to a cluster randomized controlled trial, fire stations will be randomly assigned into either intervention or control groups. No matter you are into intervention or control groups, you will receive the healthy eating information through various channels and the duration will last for 8 weeks. Regardless of which group you are in, you have to complete a set of questionnaires and measurements including body height, body weight, waist and hip circumferences, at three different time points: before you receive any intervention from us, 3 and 6 months after the completion of the intervention. The questionnaire and measurements will take you about **30 minutes** each.

The study should not result in any undue discomfort including physical or psychological aspects, you will not need to write down your name on the questionnaire. All information related to you will be kept confidential by assigning a code number for you. The raw data can only be accessed by the research team members. Your supervisor or any personnel in the Hong Kong Fire Department should not have any rights to access your information.

This is a voluntary-based study. If you returning the completed questionnaire implied that you agree to participate in this study. You have the right to skip any questions of the questionnaire if you are not willing to answer and you also have every right to withdrawn from the study before or during the study without penalty of any kind.

If you have any complaints about the conduct of this research study, please do not hesitate to contact Miss Cherrie Mok, Secretary of the Human Subjects Ethics Sub-Committee of The Hong Kong Polytechnic University in person or in writing (c/o Human Resources Office of at Room M1303 of the University).

If you would like more information about this study, please contact Ng Wing Man on telephone number 31906657 or email winnie.wm.ng@connect.polyu.hk or Dr. Kin Cheung on telephone number 27666779 or email kin.cheung@polyu.edu.hk.

Thank you for your interest in participating in this study.

Ng Wing Man
Researcher
