# Supplementary material for: A feasibility study of a WhatsApp-delivered Transtheoretical Model-based intervention to promote healthy eating habits for firefighters in Hong Kong: a cluster randomized controlled trial
Source: Trials. 2020 Jun 12;21:518. doi: 10.1186/s13063-020-04258-6 (PMC7291567; doi:10.1186/s13063-020-04258-6)
Supplement: Supplementary file 2 — Additional file 2: Informed consent form. [file 13063_2020_4258_MOESM2_ESM.pdf]

Appendix II

香港理工大學
THE HONG KONG
POLYTECHNIC UNIVERSITY

醫療及社會科學院
Faculty of Health and Social Sciences

香港 九龍 紅磡
Hung Hom Kowloon Hong Kong

CONSENT TO PARTICIPATE

A feasibility study of using various channels to promote healthy eating habits for firefighters in Hong Kong: A cluster randomized controlled trial

I, ________________ hereby consent to participate in the captioned research supervised by Dr. Kin Cheung and conducted by Ms. Ng Wing Man.

I understand that information obtained from this research may be used in future research and published. All the personal information will be coded and my right to privacy will be retained, i.e, my personal details will not be revealed.

The procedure as set out in the attached information sheet has been fully explained. I understand the benefits and risks involved. My participation in the study is voluntary.

I acknowledge that I have right to question any part of the procedure and can withdraw at any time without penalty of any kind.

Name of participant：________________

Signature of participant：________________

Name of researcher：Ng Wing Man

Signature of researcher：________________

Date：________________
