# Supplementary material for: A feasibility study of a WhatsApp-delivered Transtheoretical Model-based intervention to promote healthy eating habits for firefighters in Hong Kong: a cluster randomized controlled trial
Source: Trials. 2020 Jun 12;21:518. doi: 10.1186/s13063-020-04258-6 (PMC7291567; doi:10.1186/s13063-020-04258-6)
Supplement: Supplementary file 3 — Additional file 3: Self-administered questionnaire. [file 13063_2020_4258_MOESM3_ESM.pdf]

Reference number: ________________
Mobile number: _________________

Hong Kong firefighters' consumption of fruits and vegetables research questionnaire

Part I: Personal information

1. Age: ____________ years old

2. Gender: ☐ Male ☐ Female

3. Education level: ☐ Secondary education ☐ Community college
☐ Bachelor's degree ☐ Master's degree or above

4. Marital status: ☐ Single ☐ Married ☐ Widower
☐ Divorced ☐ Separated ☐ Live together

Part II: Working characteristics

5. Years of experiences: ______years ____ months

6. What is your current major duty? (You can choose one only)
☐ Front-line work (such as fire-fighting, emergency work, rescue work etc.)
☐ Civilian work (such as management of complaint cases etc.)
☐ Public relationship work (such as education, promotion etc.)
☐ Management work (such as coordination etc.)
☐ Others：________

7. What is your current position?
☐ Fireman ☐ Senior fireman
☐ Principal fireman ☐ Probationary station officer
☐ Station officer ☐ Senior station officer
☐ Others

8. What was/were your previous duty(ies) and working experience(s)? How long did you perform on that duty(ies)? (You can choose more than one)
☐ Front-line work (such as fire-fighting, emergency work, rescue work etc.)
_____ year(s) ___ month (s)
☐ Civilian work (such as management of complaint cases etc.)
_____ year(s) ___ month (s)
☐ Public relationship work (such as education, promotion etc.)
_____ year(s) ___ month (s)
☐ Management work (such as coordination etc.)
_____ year(s) ___ month (s)
☐ Others：________
☐ No

9. Do you need to work 24-hours shift?
☐ Yes (Please go to question 10) ☐ No (Please go to question 11)

10. Your "24-hour shift" work pattern of _____ -hour work alternating with ____ -hour off work

11. On average, how many hours do you work within a week?
___ hours

12. What is the percentage of being call out during your duty in the past 6 months?
☐ ≤ 20 % ☐ 21% - 40% ☐ 41% - 60%
☐ 61% - 80% ☐ 81% - 100% ☐ Not suitable

Part III：Eating habits
(*Last 30 days)

---

13. On average, how many days do you eat fruits within a week?
☐ No (Please go to question 16)
☐ 1 day ☐ 2 days ☐ 3 days ☐ 4 days ☐ 5 days ☐ 6 days ☐ 7 days

14. Where do you usually have fruits?
☐ At home ☐ During duty ☐ No difference, at home and during duty

15. On the day(s) that you have eaten fruit, how many fruit do you eat?
(A fruit equals to a medium-sized apple or orange, a banana, two small fruit such as kiwi fruits or plums, or half bowl of small fruit like grapes or strawberries)
☐ Less than 1 ☐ 1 - 2 ☐ More than 2

16. On average, how many days do you eat vegetables within a week?
☐ No (Please go to question 19)
☐ 1 day ☐ 2 days ☐ 3 days ☐ 4 days ☐ 5 days ☐ 6 days ☐ 7 days

17. Where do you usually have vegetables?
☐ At home ☐ During duty ☐ No difference, at home and during duty

18. On the day(s) that you have eaten vegetables, how many bowls of vegetables do you eat?
(*One bowl refers to the size of a rice bowl.)
☐ Less than 1 ☐1 - 2 ☐ More than 2

19. On average, how many days do you have breakfast (take away included) within a week?
☐ None ☐ Less than 1 ☐ 1 ☐ 2 – 4 ☐ More than 5

20. On average, how many days do you have lunch (take away included) within a week?
☐ None ☐ Less than 1 ☐ 1 ☐ 2 – 4 ☐ More than 5

21. On average, how many days do you have dinner (take away included) within a week?
☐ None ☐ Less than 1 ☐ 1 ☐ 2 – 4 ☐ More than 5

22. On average, how many days do you have night snack (take away included) within a week?
☐ None ☐ Less than 1 ☐ 1 ☐ 2 – 4 ☐ More than 5

23. How about the speed of having meal when you are on duty in the fire station when compared at home?
☐ Slower ☐ A little bit slow ☐ No difference
☐ A little bit fast ☐ Faster

Part IV：Health promotion programme on healthy eating

24. Can health promotion programme on healthy eating help you to change or understand your eating attitude and habits?
☐ Absolutely cannot ☐ Cannot ☐ Can ☐ Absolutely can

25. Can pamphlet on healthy eating alone facilitate you to change or understand your eating attitude and habits?
☐ Absolutely cannot ☐ Cannot ☐ Can ☐ Absolutely can

26. Can health promotion information on healthy eating deliver through mobile apps facilitate you to change or understand your eating attitude and habits?
☐ Absolutely cannot ☐ Cannot ☐ Can ☐ Absolutely can

Part V: Stage of Change

27. How many servings of fruits/vegetables the respondent usually consumed each day?
☐ < 2 servings for fruit and < 3 servings for vegetables (Please go to question 28)
☐ ≥ 2 servings for fruit or ≥ 3 servings for vegetables (Please go to question 29)

28. Intention to eat ≥ 2 servings a day of fruit or ≥ 3 or more servings a day of vegetables.
☐ Precontemplation (No, and I do **NOT** intend to in the **next 6 months**.)
☐ Contemplation (No, but I intend to in the **next 6 months**.)
☐ Preparation (No, but I intend to in the **next 30 days**.)
☐ Action (Yes, I have been for **LESS than 6 months**.)
☐ Maintenance (Yes, I have been for **MORE than 6 months**.)
Please go to question 31

29. Had been consuming ≥ 2 servings for fruit or ≥ 3 servings for vegetables for more than 6 months?
☐ Yes ☐ No

30. Intention to eat more?
☐ Precontemplation (No, and I do **NOT** intend to in the **next 6 months**.)
☐ Contemplation (No, but I intend to in the **next 6 months**.)
☐ Preparation (No. but I intend to in the **next 30 days**.)
☐ Action (Yes, I have been for **LESS than 6 months**.)
☐ Maintenance (Yes, I have been for **MORE than 6 months**.)

Part VI: Decisional balance and self-efficacy

Below several pros and cons of eating (fruits/vegetables) are listed below.

How important are each of these pros and cons in your decision to eat two servings of fruit each day/ three servings of vegetables each day once or twice a week?

Pros and Cons		Not at all important	Not important	Neutral	Important	Very important
31.	*Pros of fruit*					
31.1	Fruit is good for your body	☐	☐	☐	☐	☐
31.2	Other people eat fruit	☐	☐	☐	☐	☐
31.3	Fruit can substitute for unhealthy food products	☐	☐	☐	☐	☐
31.4	Eating fruits can help one maintain weight	☐	☐	☐	☐	☐
31.5	Fruit can help prevent diseases	☐	☐	☐	☐	☐
31.6	Eating fruits makes one feel better	☐	☐	☐	☐	☐
31.7	Fruits make a diet more varied	☐	☐	☐	☐	☐
31.8	Eating fruits can help one lose weight	☐	☐	☐	☐	☐
32.	*Pros of vegetable*					
32.1	Vegetables are good for your body	☐	☐	☐	☐	☐
32.2	Other people eat vegetables	☐	☐	☐	☐	☐
32.3	Vegetables can substitute for unhealthy food products	☐	☐	☐	☐	☐
32.4	Eating vegetables can help one maintain weight	☐	☐	☐	☐	☐
32.5	Vegetables can help prevent diseases	☐	☐	☐	☐	☐
32.6	Eating vegetables makes one feel better	☐	☐	☐	☐	☐
32.7	Vegetables make a diet more varied	☐	☐	☐	☐	☐
32.8	Eating vegetables can help one lose weight	☐	☐	☐	☐	☐

Pros and Cons		Not at all important	Not important	Neutral	Important	Very important
33.	*Cons of fruit*					
33.1	Eating fruits is unhandy	☐	☐	☐	☐	☐
33.2	It is hard to find tasty fruits	☐	☐	☐	☐	☐
33.3	It is difficult to store fruits	☐	☐	☐	☐	☐
33.4	Eating fruits is tasteless	☐	☐	☐	☐	☐
33.5	Eating fruits is expensive	☐	☐	☐	☐	☐
33.6	Fruit spoils quickly	☐	☐	☐	☐	☐
33.7	Recommendations for fruit are unclear	☐	☐	☐	☐	☐
33.8	It takes time to buy fruits	☐	☐	☐	☐	☐
33.9	Chemicals on fruit worry me	☐	☐	☐	☐	☐
34.	*Cons of vegetable*					
34.1	Eating vegetables is unhandy	☐	☐	☐	☐	☐
34.2	It is hard to find tasty vegetables	☐	☐	☐	☐	☐
34.3	It is difficult to store vegetables	☐	☐	☐	☐	☐
34.4	Eating vegetables is tasteless	☐	☐	☐	☐	☐
34.5	Eating vegetables is expensive	☐	☐	☐	☐	☐
34.6	Vegetables spoil quickly	☐	☐	☐	☐	☐
34.7	Recommendations for vegetables are unclear	☐	☐	☐	☐	☐
34.8	It takes time to prepare vegetables	☐	☐	☐	☐	☐
34.9	It is difficult to prepare vegetables	☐	☐	☐	☐	☐
34.10	Chemicals on vegetables worry me	☐	☐	☐	☐	☐

35. Do you think it is easy or difficult to eat two servings of fruit each day/ three servings of vegetables each day once or twice a week?

Self-efficacy		Very difficult	Difficult	Neutral	Easy	Very easy
35.1	*Self-efficacy for fruit*					
35.1.1	In the weekends	☐	☐	☐	☐	☐
35.1.2	During working days	☐	☐	☐	☐	☐
35.1.3	In winter	☐	☐	☐	☐	☐
35.1.4	When you don't have much time	☐	☐	☐	☐	☐
35.1.5	When you experience (emotional) distress	☐	☐	☐	☐	☐
35.1.6	When having a touch of flu or a cold	☐	☐	☐	☐	☐
35.2	*Self-efficacy for vegetable*					
35.2.1	In the weekends	☐	☐	☐	☐	☐
35.2.2	During working days	☐	☐	☐	☐	☐
35.2.3	In winter	☐	☐	☐	☐	☐
35.2.4	When you don't have much time	☐	☐	☐	☐	☐
35.2.5	When you experience (emotional) distress	☐	☐	☐	☐	☐
35.2.6	When having a touch of flu or a cold	☐	☐	☐	☐	☐
