# Supplementary material for: A feasibility study of a WhatsApp-delivered Transtheoretical Model-based intervention to promote healthy eating habits for firefighters in Hong Kong: a cluster randomized controlled trial
Source: Trials. 2020 Jun 12;21:518. doi: 10.1186/s13063-020-04258-6 (PMC7291567; doi:10.1186/s13063-020-04258-6)
Supplement: Supplementary file 4 — Additional file 4: Record of measurements. [file 13063_2020_4258_MOESM4_ESM.pdf]

Record of measurements

Name: _______________ Reference number: _________________

	Baseline (T_0)	3 months (T_1)	6 months (T_2)
Date			
Body height (cm)			
Body weight (kg)			
Body mass indexes (BMI) (kg/m^2)			
Waist circumference (cm)			
Hip circumference (cm)			
Waist-to-hip ratios (WHR)			

References:

BMI (kg/m^2)	Asia
Underweight	< 18.5
Normal	18.5 – 22.9
Overweight	23.0 – 24.9
Moderately obese	25.0 – 29.9
Severely obese	≥ 30.0

WHR	Asia (male)
Normal	0.90
